# Wild Species from the Family Apiaceae, Traditionally Used as Food in Some Mediterranean Countries

**DOI:** 10.3390/plants13162324

**Published:** 2024-08-21

**Authors:** Ekaterina Kozuharova, Giuseppe Antonio Malfa, Rosaria Acquaviva, Benito Valdes, Alla Aleksanyan, Daniela Batovska, Christina Stoycheva, Moh Rejdali, Abdel Rahman Al-Tawaha, Pasquale Marino, Vivienne Spadaro

**Affiliations:** 1 Department of Pharmacognosy, Faculty of Pharmacy, Medical University of Sofia, 1000 Sofia, Bulgaria; christina9828@gmail.com; 2 Department of Drug and Health Sciences, University of Catania, Viale A. Doria 6, 95125 Catania, Italy; gmalfa@unict.it (G.A.M.); racquavi@unict.it (R.A.); 3 Research Centre on Nutraceuticals and Health Products (CERNUT), University of Catania, Viale A. Doria 6, 95125 Catania, Italy; 4 Department of Plant Biology and Ecology, University of Seville, Avda. Reina Mercedes s/n, 41012 Seville, Spain; bvaldes@us.es; 5 Department of Geobotany and Ecophysiology, Institute of Botany aft. A. Takhtajyan NAS RA, Acharyan 1, Yerevan 0063, Armenia; alla.alexanyan@gmail.com; 6 Institute of Chemical Engineering, Bulgarian Academy of Sciences, Acad. Georgi Bonchev Str., Bl. 103, 1113 Sofia, Bulgaria; danielabatovska@gmail.com; 7 Departement de l’ Environement, Institut Agronomique et Vétérinaire Hassan II, Rabat 10112, Morocco; m_rejdali@hotmail.com; 8 Department of Biological Sciences, Al-Hussein Bin Talal University, Ma’an P.O. Box 20, Jordan; abdeltawaha74@gmail.com; 9 PLANTA/Center for Research, Documentation and Training, Via Serraglio Vecchio 28, 90123 Palermo, Italy; marino@centroplantapalermo.org; 10 Department of Biological, Chemical and Pharmaceutical Sciences and Technologies, Section of Botany, Anthropology and Zoology, University of Palermo, Via Archirafi 38, 90123 Palermo, Italy; vivenne.spadaro@unipa.it

**Keywords:** consumed wild umbellifers, similarity, distribution, Jaccard index, heatmap clustering

## Abstract

Mediterranean countries are a cauldron of cultural exchange, with a strong emphasis on wild plants in cuisine traditions. Many of these plants belong to the family Apiaceae. The common climate determines the common range of distribution. While many plants have wide distribution, the range of distribution of others is restricted to Western Mediterranean or North Africa. This review investigates wild plants from the family Apiaceae traditionally used as food in 13 study sites—11 countries in the Mediterranean and adjacent territories—the mainland and 3 islands. The aim is to trace patterns of native distribution versus consumption. As a result, 81 wild umbellifers are listed, traditionally used as food. Their consumption and distribution patterns are described and discussed. Interestingly in 8 of the 13 study sites (61.5%) are recorded 50 plant taxa (66.7% of all wild umbellifers, traditionally used as food) which are consumed in only one particular country. These are as follows: 8 taxa in Morocco, 9 taxa in Spain, 2 taxa in Sicily, 3 taxa in Bulgaria 3 taxa in Crete, 8 taxa in Armenia, 14 taxa in Turkey, and 3 taxa in Jordan. However, these 50 restrictedly consumed plants are distributed in more than one country (except 15 taxa, which are endemics). Many of the plants that are used in certain countries are not consumed by the neighboring people. The results of the two statistical tests, namely Jaccard index and heatmap clustering (double dendrogram), are discussed. The presence of an outlier, such as Bulgaria, which shares borders, history, and culture with Greece and Turkey, emphasizes the importance of local climate for plant distribution and consumption over cultural interactions. The same was observed for some pairs of countries, such as Spain and Morrocco and Turkey and Armenia, although they had the highest number of common plants that are both distributed and consumed as food.

## 1. Introduction

The use of wild edible plants is linked to the cultural history of a certain region, the local identity, and traditions of the populations. The Mediterranean basin is a cauldron of different cultures and religious beliefs, which, over the centuries, have been mixed together due to great migrations of populations or to invasions and conquests. For instance, Muslim invaders conquered many European countries in the Middle Ages (10th and 11th centuries) [1]. The Ottoman Empire expanded in 14th and 15th century—westwards on the territory of the Balkan peninsula, southwards to the Near East and North Africa, and eastwards to the Caspian Sea including the territory of Armenia [2]. Also, cultural relations can be expected between the eastern part of Spain and Sicily, as the island was part of the Aragon Kingdom in the 13th century. Moreover, Sicily was able to resist the demands of the maritime cities, namely Pisa and Genoa, and preserve a strong political core [3]. Cultural intersections are also known for Spain and Morocco in the Middle Ages [4].

The commercial trade across the Mediterranean Sea facilitated the exchange of knowledge between the different cultures that surrounded this area, including the use of wild plants as food. Thanks to Mediterranean traditions, a large number of them continue to be present in the food [5]. Native plants have been our most immediate source of food since ancient times. People all over the world experimented with and ate different parts of the plants they found growing locally. Many of these were subjected to continuous selection and domestication processes over long periods of time, and as populations migrated to new territories, the plants also moved with them [6]. The result is a variety of eating styles with many common elements, but, at the same time, they differ in distinct local or regional traditions.

The wild plants consumed by Mediterranean people have been the subject of interest since antiquity for their beneficial effects on human health. The relative longevity of these people is attributed to their diet. Indeed, the Mediterranean diet is considered a healthy and sustainable food model, combining respect for the biodiversity, creating a strong sense of cultural identity, and continuity of these communities in the Mediterranean basin [7]. In Mediterranean countries, the traditional diet is usually based on the eating of a large number of plants collected from nature, including climbing plants with young edible sprouts, wild tubers and leafy vegetables, edible fruits of tree species, as well as aromatic plants used as spices [8,9]. There has been increased interest in edible wild plants in recent years, and the knowledge of their phytochemicals in the role of health care allows them to be called “new functional foods” [10].

About 2300 different fungi and plants are collected from nature as food in the Mediterranean region, many of these being umbellifers. For example, 336 wild plant taxa are collected and consumed in Andalusia (7% of the total wild flora). Nearly 6% of these, or 20 plant species, are umbellifers [11]. The Apiaceae family consists of about 3780 species in 434 genera, most of which are native to the Mediterranean region and South-Western Asia. It includes a large number of wild umbellifers used for different purposes since ancient times (food, traditional medicine) as well as in modern pharmaceutical and cosmetic industries [12,13].

The common Mediterranean climate determines the common range of distribution of many plants. While plenty of them grow all over the territory, some occur restrictedly either to the Western or to the Eastern Mediterranean. Some plants grow only in North Africa, while others do not reach so much to the South [14]. Thus, floristic specifics are the precondition for some plants to be used as food in some regions/countries and not in others.

**Figure 1:**
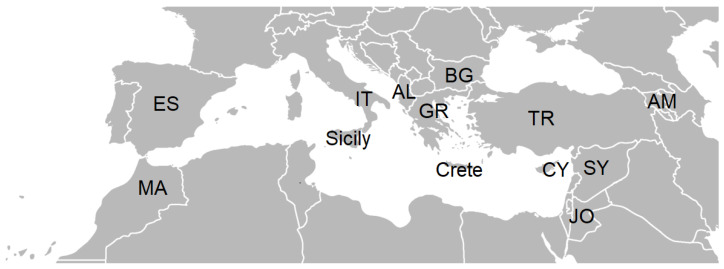
Study sites. Legend: JO—Jordan and Palestine; IT—Southern Italy: MA—Morocco; Sicily; ES—Spain; SY—Syria; TR—Turkey (Türkiye); AL—Albania; AM—Armenia; BG—Bulgaria; Crete; CY—Cyprus; GR—Greece. Credit https://commons.wikimedia.org/wiki/File:BlankMap-Europe-v4.png, accessed on 4 July 2024.

The aim of this review is to study wild plants from the family Apiaceae traditionally used as food in 13 study sites—11 countries, including 3 islands in the Mediterranean and adjacent territories (Figure 1)—in order to trace patterns of native distribution and cultural exchange. The hypothesis is that countries that are neighboring will have high similarity in (1) distribution of umbellifer taxa and (2) the use as food of wild umbellifers.

## 2. Results

### 2.1. Distribution of Wild Umbellifers Traditionally Used as Food

As a result of our literature survey, we derived a list of 81 wild umbellifers traditionally used as food in 13 study sites—the mainland and 3 islands of 11 countries in the Mediterranean and adjacent territories (Figure 1, Table 1).

The distribution of these 81 taxa is either all over the territory of the studied sites or restricted to certain parts (Table 1).

**Figure 2:**
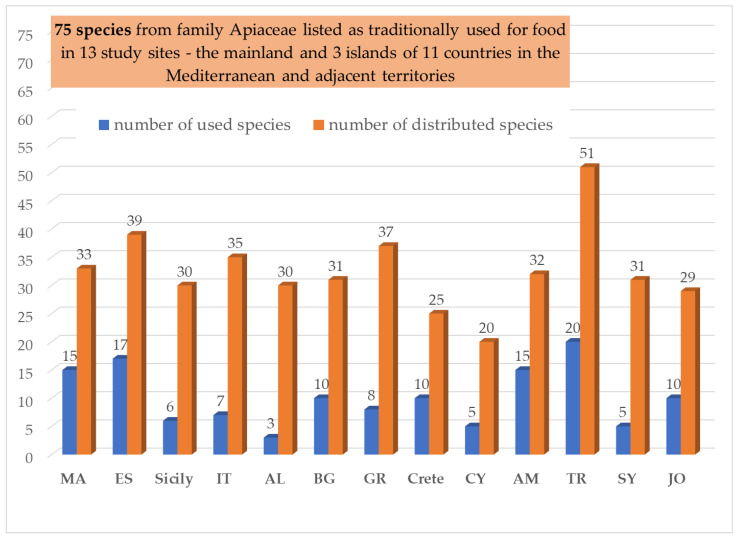
Number of wild plants from the family Apiaceae used as food and distributed in each of the study sites.

The listed 81 wild umbellifers traditionally used as food (Table 1) are reduced to 75 taxa suitable for statistically analyses (as explained in the Section 4). These 75 taxa are distributed as follows: 15 taxa in Morocco [15,16,17,18,19,20,21,22], 17 taxa in Spain [23,24,25], 6 taxa in Sicily [26,27,28,29], 7 taxa in Southern Italy [30,31,32,33,34], 3 taxa in Albania [32,34,35,36], 8 taxa in Greece [37,38], 10 taxa in Crete [37,39,40], 5 taxa in Cyprus [37,40], 20 taxa in Turkey (Türkiye) [41,42,43,44,45], 10 taxa in Bulgaria [42,46,47,48,49,50], 15 taxa in Armenia [51,52,53,54,55,56,57,58], 5 taxa in Syria [59], and 10 taxa in Jordan and Palestine [60,61,62,63] (Figure 2).

The highest number of edible wild umbellifers is reported for Turkey, followed by Spain (n = 17), Armenia (n = 15), and Morocco (n = 15) (Figure 2). A similar pattern is seen in the distribution—Turkey, followed by Spain, Greece, Italy, Morocco, and Armenia (Figure 2). 

**Figure 3:**
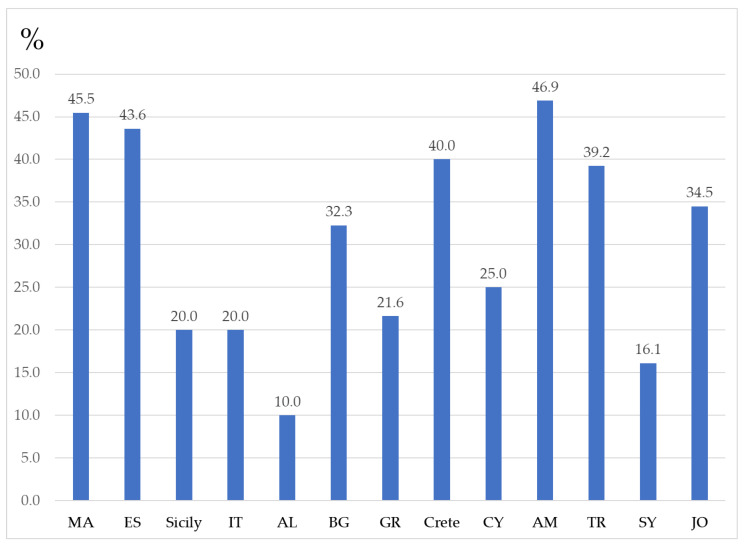
Percentage of traditionally consumed wild umbellifers of all distributed edible umbellifers in each country.

It is also shown that only about half or even less of the distributed edible umbellifers are consumed traditionally in each country (Figure 3). This indicates that the knowledge does not cross the borders that much as expected, because many of the wild plants that are used in a certain country are not used by the neighbors. Armenia holds the record of utilization of wild edible umbellifers distributed on its territory (46.9%), followed by Morocco (45.5%), Spain (43.6%), Crete (40.0%), and Turkey (39.2%). Interestingly, Turkey is home to the highest number (51 taxa) of the umbellifers recognized as edible (68%), but only 39.2% of them are used as food. Additionally, 14 taxa (70% of all wild umbellifers used as food in Turkey) are consumed only in Turkey. The lowest consumption is observed in Albania—only 10.0% of the of wild edible umbellifers distributed on its territory are utilized.

**Figure 4:**
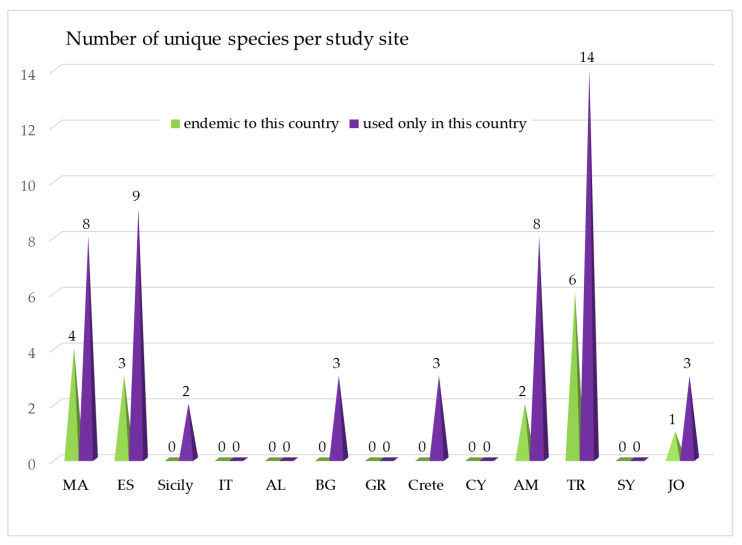
Number of wild plants from the family Apiaceae with limited use as food and distributed in each of the study sites.

It is notable that 50 plants are used as food only in one particular country, although most of them are distributed in several other countries. They represent 66.7% of all wild umbellifers traditionally used as food in the studied sites. Such unique consumption is recorded in eight of the study sites (61.5% out of all study sites). The distribution is as follows: 8 taxa are used as food only in Morocco, 9 taxa in Spain, 2 taxa in Sicily, 3 taxa in Bulgaria 3 taxa in Crete, 8 taxa in Armenia, 14 taxa in Turkey. and 3 taxa in Jordan and Palestine (Figure 4).

Some of the wild traditionally consumed umbellifers are endemic and have restricted distribution to only one or few neighboring countries. Limited is the distribution of 16 taxa (Figure 4). They comprise20% of all documented umbellifers used as food. These are as follows: 5 taxa endemic to Turkey—*Diplotaenia turcica* Pimenov & Kljuykov (syn. *D. cachrydifolia* non Boiss.), *Chaerophyllum macropodum* Boiss., *Ferulago angulata* (Schlecht.) Boiss., *F. stellata* Boiss.; 1 taxon endemic to Turkey and Armenia—*Grammosciadium platycarpum* Boiss. & Hausskn. (but it is used only in Turkey); 3 taxa endemic to Spain—*Bunium balearicum* (Sennen) Mateo & López Udías, *Conopodium subcarneum* (Boiss. & Reut.) Boiss. & Reut., and *C. thalictrifolium* (Boiss. & Reut.) Boiss. & Reut.; 1 taxon endemic to Morocco*—Anethum foeniculoides* Maire & Wilzek; 1 taxon in Armenia—*Angelica tatianae* Bordz, endemic to North Caucasus, Georgia, and Azerbaijan; 1 taxon distributed in Jordan and a few neighbor countries—*Deverra tortuosa* (Desf.) DC. The other 3 taxa are plants growing only in North Africa (Morocco and neighboring countries are *Deverra scoparia* Coss. & Durieu, *D. denudata* (Viv.) Pfisterer & Podlech, *Ammodaucus leucotrichus* Coss. & Durieu etc.) (Figure 4, Table 1).

Additionally, the range of *Conopodium marianum* Lange, *C. pyrenaeum* (Loisel.) Miégev, *Bunium pachypodum* P.W. Ball etc., (Table 1), is restricted to Western Mediterranean.

### 2.2. Statistical Analyses of the Distribution and Consumption of Wild Umbellifers

#### 2.2.1. Jaccard Index

The Jaccard index (JI) revealed that the level of similarity between pairs of countries in traditional use of wild umbellifers as food is rather different regarding the distribution pattern of edible wild umbellifers in the same pairs of countries there.

**Figure 5:**
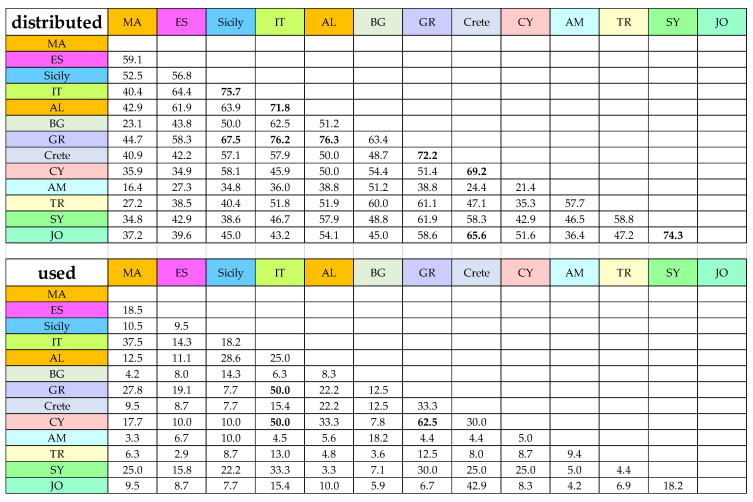
Similarity between pairs of countries in both distribution of edible wild umbellifers and traditional use of wild umbellifers as food, expressed as JI %. The numbers above 65% for the similarity of distribution and above 50% for the similarity of use are marked in bold for convenience.

The highest similarity regarding the distribution pattern of edible wild umbellifers is between the pair of Albania and Greece, immediately followed by Italy and Greece, Sicily and Italy, Syria and Jordan, Greece and Crete, and Italy and Albania (Figure 5).

The pairwise similarity regarding the pattern of consumption differs from the distribution pattern. The values of JI are lower for all the pairs. The highest similarity regarding the use pattern of wild umbellifers is between Greece and Cyprus, followed by the pairs Southern Italy and Greece, Southern Italy and Cyprus, Crete and Jordan and Palestine, Morocco and Southern Italy, Greece and Crete, Greece and Syria, Crete and Cyprus, etc. (Figure 5). Contrary to the expected high similarity in use pattern between Bulgaria and Turkey, both neighboring countries and parts of the Ottoman Empire, the JI value is low. Also, a low JI value is calculated for the pair of Turkey and Greece, both neighboring countries and parts of the Ottoman Empire. Additionally, the use pattern similarity between Morocco and Spain is not high, on the contrary of the expected (Figure 5).

#### 2.2.2. Heatmap Clustering

The heatmap (double dendrogram) highlights the similarities between all of the plants and countries studied in terms of Apiaceae wild plant distribution/food consumption patterns (Figure 5). The patterns are divided into three categories: not distributed and not used as food, distributed, but not used as food, and both distributed and used as food. The columns of the heatmap depict the similarities between patterns per country, while the rows represent the plants.

**Figure 6:**
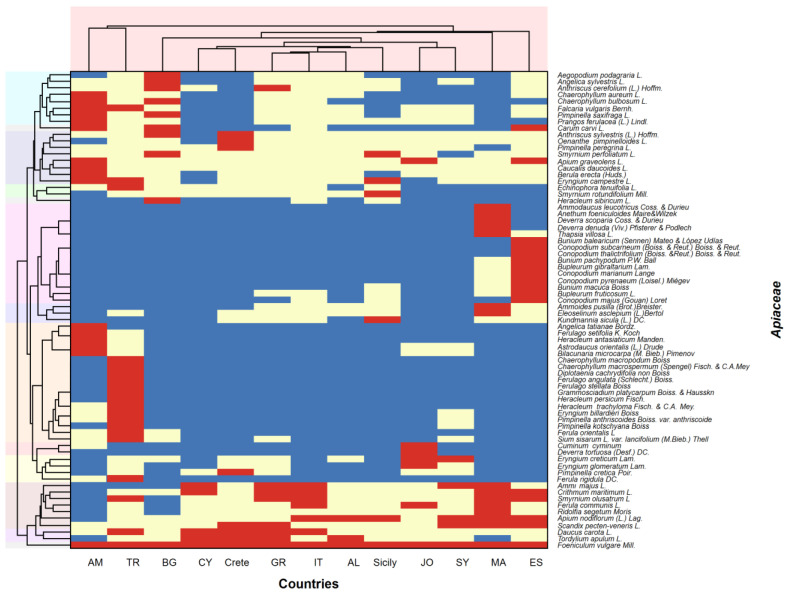
Heatmap and dendrogram showing clustering of wild-growing Apiaceae plants with similar distribution and eating pattern across the studied countries and clustering of countries with similar distribution and eating pattern of the same plants. The colors used refer to specific patterns as follows: blue—not distributed and not used as food, yellow—distributed, but not used as food, and red—distributed and used as food.

Countries cluster together according to their geographical proximity. Thus, Armenia is linked with Turkey, whereas Morocco is associated with Spain (Figure 6). The remaining countries are clustered together as follows: the islands of Crete and Cyprus; Greece and Italy, as well as the more distant members Albania and Sicily; Jordan and Syria. Bulgaria is something of an outlier, despite having common borders with Turkey and Greece. In the pattern where plants are both distributed and consumed as food, the greatest parallels can be seen between Morocco and Spain, Greece and Italy, Crete and Cyprus, and Armenia and Turkey (Figure 6).

Plant clusters that deserve attention comprise the following: *Angelica tatianae* Bordz, *Ferulago setifolia* K. Koch, *Heracleum antasiaticum* Manden., *Astrodaucus orientalis* (L.) Drude, *Cachrys microcarpa* M. Bieb. (syn. *Bilacunaria microcarpa* (M. Bieb.) Pimenov) and *Chaerophyllum macropodum* Boiss., *Chaerophyllum macrospermum* (Spengel) Fisch. & C. A. Mey., *Diplotaenia turcica* Pimenov & Kljuykov (syn *D. cachrydifolia* sensu P.H. Davis, non Boiss.), *Ferulago angulata* (Schlecht.) Boiss., *Ferulago stellata* Boiss., *Grammosciadium platycarpum* Boiss. & Hausskn., *Heracleum persicum* Fisch., *Heracleum trachyloma* Fisch. & C.A. Mey. *Eryngium billardieri* F. Delaroche, *Pimpinella anthriscoides* Boiss., *Pimpinella kotschyana* Boiss., *Ferula orientalis* L., *Sium sisarum* L., respectively used either only in Armenia or only in Turkey (Figure 6). 

The next cluster of plant groups is those used in Morocco (*Ammodaucus leucotrichus* Coss. & Durieu, *Deverra scoparia* Coss. & Durieu, *D. denudata* (Viv.) Pfisterer & Podlech, *Thapsia villosa L.*) and associated with Spain (*Bunium balearicum* (Sennen) Mateo & López Udías *B. macuca* Boiss., *B. pachypodum* P.W. Ball, *Bupleurum fruticosum* L., *B. gibraltaricum* Lam., *Conopodium marianum* Lange, *C. pyrenaeun* (Loisel.) Miégev, *Conopodium majus* (Gouan) Loret) (Figure 6).

*Scandix pecten-veneris* L., *Daucus carota* L., and *Tordylium apulum* L. group in a cluster which is related to the consumption of these plants in Greece, Crete, and Cyprus, but also in some other countries (Figure 6).

## 3. Discussion

The consumption of wild umbellifers is basically well documented in all studied countries (Table 1).

The only plant that is reported “consumed wild” in all countries is *Foeniculum vulgare* L. Additionally, it is an important commercial vegetable for its swollen, bulb-like stem base, and varieties are cultivated widely [64,65].

Some interesting cases that deserve special comments are *Anethum graveolens* L., *Petroselinum crispum* (Mill.) Fuss, *Cuminum cyminum* L., and *Apium graveolens* L. (Table 1, Figure 6). These are, respectively, dill, parsley, cumin, and celery, which are quite popular spices [66].

Dill, *Anethum graveolens,* which is reported among the wild umbellifers used as food in Turkey and Jordan, is native to Turkey and Jordan but also to Albania, Bulgaria, Cyprus, Syria, Morocco, and Spain [14] (only study sites where the plant is native are taken into account in the data set for the calculation of the Jaccard index). This plant was introduced to Armenia, Crete, and Greece. Interestingly it appears in the reports of wild umbellifers used in Jordan (Table 1) [62]. Dill is naturalized in Italy and casual to Sicily and Spain [14]. Dill is widely used in all countries, being a very popular spice, which is vastly cultivated. For example, it is a very popular food plant, cultivated commercially and in kitchen gardens in Bulgaria. It is not reported among the wild umbellifers used as food in this country. *A. graveolens* is a wild but rare plant there (category “Endangered” according to the Red Data Book) [67].

The well-known and widely used parsley, *Petroselinum crispum* (Mill.) Fuss, is reportedly consumed from wild in Jordan. It is a native plant there, as well as in Morocco and Albania. Parsley is casual to Sicily and introduced to Armenia, Bulgaria, Greece, Turkey, Crete, and Cyprus and naturalized to Spain [14].

Commercially grown and widely used cumin, *Cuminum cyminum* L., is native to the Irano-Turanian Region [68], including Jordan and Armenia (Table 1). It is consumed wild in Jordan [62].

The commercially cultivated celery, *Apium graveolens* L., which is native to Albania, Bulgaria, Crete, Cyprus, Greece, Jordan, Italy, Morocco, Sicily, Spain, Syria, and Turkey, is consumed wild in Jordan and some provinces of Spain. Interestingly, it is mentioned as used as food from the wild in Armenia, but it is considered “introduced” to this country (therefore, this particular record was eliminated from the statistical analysis). The reported consumption of popular and commercially cultivated spices from wild in Jordan indicates this part of the world as a center of domestication of dill, parsley, and cumin, and quite an ancient one [69].

Ferula is a key symbolic plant, known from antiquity and even before that (Levey, M. (1958)) [70]. Ferula is associated with the production of perfume in Minoan civilization [71]. Unidentified Ferula (*Ferula assa-foetida* L., or *F. orientalis* L.) images are found on ancient Greek coins [72]. *F. communis* L. has been an important plant in Sicily during the antiquity [73]. Different species of the genus are used as food only in Jordan, Turkey, Morocco, and Southern Italy (Table 1), although particularly *F. communis* L. is distributed not only in these countries, but also in Crete, Cyprus, and Greece.

The Jaccard index is a widely used method in ecology to evaluate pairwise similarity of two groups considering the presence versus absence of members. It gives us a possibility to compare the similarity between pairs of countries firstly by the distribution of umbellifers taxa and secondly, but separately, by the consumption of these plants. This approach provides interesting and sometimes unexpected details of our study. For example, the similarity between the pairs of Southern Italy and Greece and Southern Italy and Cyprus is higher than Southern Italy and Sicily. The heatmap approach regards simultaneously both distribution and consumption patterns in a different angle of view. These two models are not contradictive but complementary. The JI reveals similarity differences in some pairs of countries regarding the distribution and the consumption (Figure 5). The heatmap reveals that countries cluster together according to their geographical proximity. It visualizes, for example, the big differences in distribution patterns between the distant regions such as Turkey and Armenia versus Morocco and Spain. At the same time, tracing the color coding, it is notable that within the country clusters, certain plants are used as food in some countries while in others (neighboring) they are present but not consumed (Figure 6). In addition, it groups together plants used in certain countries.

The knowledge about edible plants is precious heritage that needs to be preserved and spread among the people. This way it can contribute to the introduction of culture, sustainable development and biodiversity conservation. A bridge between the generations in the digital era is needed for these efforts. Such a tool is media pedagogy [74].

## 4. Materials and Methods

The object of our study is the wild plants from the family Apiaceae traditionally used as food on the territory of 12 countries (mainland and 3 islands, Figure 1) in the Mediterranean and adjacent territories. We followed the principles for historical unity (e.g., Roman Empire, Byzant Empire, Visigothic Kingdom, Ostgothic Kingdom, Umayyad Caliphate Ottoman Empire, Aragon Kingdom, The Kingdom of Sicily, etc.) [75] and the potential for cultural exchange, territorial neighborhood, and phytoclimatic and floristic relationships.

We accessed Google Scholar, Web of Science, and PubMed to identify publications for the period 1990–2022 using the search string: “Spain”, “Morocco”, “Sicily”, “Italy”, “Albania”, “Greece”, “Crete”, “Cyprus”, “Turkey”, “Bulgaria”, “Armenia”, “Egypt”, “Syria”, “Jordan and Palestine”, “Kosovo”, North Macedonia” as well as “traditionally” “wild”, “food”, “plants”, “ethnobotany”, etc. These countries were selected according to the territorial neighborhoods but also historical cultural influences.

Following the PRISMA 2000 guidelines [76], the records were assessed for eligibility. In total, 452 papers were excluded either because (1) the information was not in accordance with the topic of this research; (2) the data on the traditional use were only about medicinal purposes; (3) the studies analyzed traditional food habits of the local populations but did not provide information about wild plants; (4) the records were for consumption of plants that were cultivated.

### 4.1. Distribution of Wild Umbellifers Traditionally Used as Food

From the selected publications, we extracted the information about the wild plants from the family Apiaceae traditionally used as food in Spain [23,24,25], Morocco [16,17,18,19,20,21,22], Sicily [26,27,28,29], Southern Italy [30,31,32,33,34], Albania [32,34,35,36], Greece [37,38], Crete [37,39,40], Cyprus [37,40], Turkey (Türkiye) [41,42,43,44,45], Bulgaria [42,46,47,48,49,50], Armenia [51,52,53,54,55,56,57,58], Syria [59], and Jordan and Palestine [60,61,62,63]. We found that there are not enough studies and publications on the wild plants traditionally used as food in several Mediterranean countries such as France, Egypt, Kosovo, North Macedonia, etc., providing sufficient data for the aim of this investigation.

### 4.2. Data Set Preparation and Analyses

We organized the reported data for each country in Excel tables. The range of distribution of each plant taxon on the territories of the studied countries was added in the tables following [14]. Some mistakes of the published data were noticed and eliminated in the tables and analyses. For example, *Oenanthe javanica* (Blume) DC., *Ostericum sieboldii* (Miq.) Nakai, and *Ferula sinkiangensis* K.M. Shen reported for Jordan and Palestine [62] are plant taxa distributed in the Far East but not in the Near East. Therefore, they are not included in Table 1 and the analysis. Also, *Pimpinella anisum* L. is not included in Table 1 and the analysis. This plant is reported among the umbellifers used from the wild in Jordan and Palestine [61]. However, it is cultivated, introduced, naturalized, or casual in all studied countries but nowhere wild [14]. Additionally, *Bunium macuca* Boiss., reported to be used in Jordan and Palestine, is not distributed in this country because it is a west Mediterranean taxon. It is considered only for Spain in Table 1 and in the analyses of the use, but not for Jordan.

Particular taxa are casual (alien species that do not form self-sustaining populations in the invaded region) or naturalized plants in some countries, and these cases are discussed in the text with caution. For example, this is the case with lovage, *Levisticum officinale* W.D.J. Koch, which is often grown in herb gardens. It is native to Iran and Afghanistan [14] but not to Jordan, although it is reported to be consumed wild in this country. This taxon is excluded from the statistical analysis. The records about 6 wild umbellifers traditionally used as food in our study sites are found to have peculiarities. *Coriandrum sativum* L., which is reported among the wild umbellifers used as food in Armenia [51,52,53,54,55,56,57,58] and Turkey [41,42,43,44,45], is native only to Jordan and Syria but introduced to Armenia, Turkey, Bulgaria, Albania, Greece, Crete, and Cyprus [14]. This worldwide popular spice is also naturalized to Italy and Morocco and casual to Sicily and Spain [14]. *Ferula assa-foetida* L. is reported to be consumed as a wild plant in Jordan, but it is native only to Libya [14]. The same wild consumption is reported about *Centella asiatica* (L.) Urb. in Jordan, which, however, is native only to Georgia.

*Pastinaca sativa* L. is a popular commercially cultivated vegetable in Europe. It is native to Albania, Bulgaria, Greece, Italy, Sicily, Spain, Turkey, and Armenia. However, there are no reports for gathering from the wild in these countries nowadays. At the same time, such use is reported about Jordan, where this plant is not distributed (Table 1). These peculiarities of the records made them not suitable for the statistical analyses, and we excluded them from the calculations.

In the analyses, wild carrot was considered to a species level, although it was specified that in Spain and Jordan, it was *Daucus carota* L. subsp. *maximus* (Desf.) Ball Jazar Barri, while in Sicily, it was the typical subspecies, but in the other countries the subspecies level is not mentioned.

We marked which plant part was used as well as the way of consumption. The information provided in the original sources was transcribed (Table 1). It was not possible to standardize the use of plant parts because the descriptions of consumption ways were not equally precise and detailed in the original publications. Due to that, the data matrix that we obtained was not suitable for a detailed analysis of both convergent and divergent use of plants and further cultural exchange interpretations. Ethnobotanists sometimes provided synonyms in the published original sources and a replacement with the accepted names was performed following [14]. They were used to perform basic descriptive statistics.

#### 4.2.1. Jaccard Similarity Coefficient or Jaccard Index (JI)

The Jaccard similarity coefficient or JI is used when the level of similarity between two groups of elements should be identified [77]. We used the JI to evaluate the similarity of use and similarity of distribution among all possible country pairs. The JI is calculated using the following formula:

JI [%] = N_AB_ × 100/(N_A_ + N_B_ − N_AB_)

where

N_A_ is the number of elements in study site A (country/mainland or island),
N_B_ is the number of elements in study site B (country/mainland or island),
N_AB_ is the number of elements available in both study sites (country/mainland or island).

#### 4.2.2. Heatmap Clustering

The clustered heatmap (double dendrogram) was created using the Group Average (Unweighted Pair-Group) Clustering Method and Euclidean distance in NCSS 24.0.2 Statistical Software [78].

## 5. Conclusions

This work, focused on the wild plants of the Apiaceae family traditionally used as food in some Mediterranean countries, has provided an overview of the most commonly consumed umbellifers, highlighting similarities and differences between the territories of reference. In particular, this survey revealed 81 specific and infraspecific taxa from the Apiaceae family used traditionally as food in 11 Mediterranean countries and 3 islands. This also makes it possible to pass on to future generations traditional knowledge about wild edible plants, which, in the particular context of the Mediterranean diet, is of considerable relevance. The similarities of wild umbellifers consumption are found in geographical patterns based on plants’ range of distribution and cultural exchange. The working statement that neighboring countries have high similarity in the distribution pattern of edible wild umbellifers is confirmed by both the Jaccard index and heatmap clustering statistical models. The wild umbellifers consumption does not follow strictly the distribution pattern. Both models indicate the important role of the cultural exchange, but tracing it is too complicated and impossible.

## Figures and Tables

**Table 1:** Wild plants from the family Apiaceae traditionally used as food and their distribution in 11 Mediterranean countries and adjacent territories. Legend: JO—Jordan and Palestine; IT—Southern Italy; MA—Morocco; Sicily; ES—Spain; SY—Syria; TR—Turkey; AL—Albania; AM—Armenia; BG—Bulgaria; Crete; CY—Cyprus; GR—Greece. Note: asterisks mark those species that are collected in the field and eaten only in 1 or 2 of the 50 Spanish provinces, while all other species listed for Spain are used in 3 or more provinces. Distribution of the taxa basically follows Euro + Med-Plantbase [14], double asterisks mark the synonym names used in the original publications.

Taxon	Presence in Countries of the Study Area	Used as Food	Used Part and Modes of Application
*Aegopodium podagraria* L.	AL, BG, ES, GR, IT, TR	BG	Sprouts, salad raw, soup
*Ammi majus* L.	AL, Crete, CY, GR, ES, IT, JO, MA, Sicily, SY,	SY	Inflorescence, added to yerba mate
		CY	Aerial parts, salads
		GR	Aerial parts, sauté
		IT	Aerial parts boiled then on pasta, dressing with “pecorino” cheese and olive oil
		MA	Stems
*Ammodaucus leucotrichus* Coss. & Durieu	MA	MA	Seeds
*Ammoides pusilla* (Brot.) Breister.	AL, ES, GR, IT, MA, Sicily	MA	Aerial parts
*Anethum foeniculoides* Maire & Wilzek	MA	MA	Aerial parts, seeds
*Anethum graveolens* L.	native AL, BG, CY, ES, MA, TR, introduced to AM, Crete, GR, JO, SY; naturalized in IT, casual to Sicily;	TR	Aboveground, salad, seasoning, raw, plant is consumed with yogurt
		JO	Seed Consumed cultivated in BG, as the plant is listed as Endangered in the Red Data Book
*Angelica sylvestris* L.	AL, AM, BG, ES, GR, IT, Sicily, SY, TR	BG	Sprouts, leaves, salad raw, soup
*Angelica tatianae* Bordz.	AM	AM	Petiole, stem
*Anthriscus cerefolium* (L.) Hoffm.	native AM, AL, BG, CY, GR, Sicily, TR introduced to MA, IT, naturalized to ES	BG	Leaves, shoots, salad raw, soup, spice
		GR	Young aerial parts, boiled
*Anthriscus sylvestris* (L.) Hoffm.	AL, AM, BG, ES, GR, IT, JO, MA, Sicily, SY, TR	AM	Stems, leaves
		BG	Roots, leaves, shoots, salad raw, soup, pastry
		Crete	Young aerial parts, fried in mixtures as a filling for pies
*Apium graveolens* L.	native AL, AM, BG, Crete, CY, ES, GR, IT, JO, MA, Sicily, SY, TR	JO	Petiole, leaves
		AM	Leaves, roots
		* ES	Leaves, raw in salads or stewed
*Astrodaucus orientalis* (L.) Drude	AM, JO, SY, TR	AM	Stems
*Berula erecta* (Huds.) Coville	AL, AM, BG, Crete, ES, GR, IT, JO, MA, SY, TR	AM	Leaves, fruits
*Bunium balearicum* (Sennen) Mateo & López Udías	ES	* ES	Tubers, raw as a snack
*Bunium macuca* Boiss.	ES, MA, Sicily	ES	Tubers, raw as a snack (they taste like chestnut)
*Bunium pachypodum* P.W. Ball	ES, MA	* ES	Tubers, raw as a snack
*Bupleurum fruticosum* L.	ES, GR, IT, MA, Sicily	* ES	Aerial parts for “hardening” olives (the fresh fruits, which are not yet edible, are placed in brine to improve the taste)
*Bupleurum gibraltaricum* Lam.	ES, MA	* ES	Aerial parts for “hardening” olives (the fresh fruits, which are not yet edible are placed in brine to improve the taste)
*Cachrys microcarpa* M. Bieb. (syn. ** *Bilacunaria microcarpa* (M. Bieb.) Pimenov & V.N. Tikhom.	AM, JO, SY, TR	AM	Stems, leaves
*Carum carvi* L.	AL, AM, BG, ES, IT, TR	AM	Stems, leaves, seeds
		BG	Fruits, spice
		* ES	Fruits
*Centella asiatica* (L.) Urb.	native in Georgia and tropical and subtropical Asia and Africa	JO	Aboveground organs
*Chaerophyllum aureum* L.	AL, AM, BG, ES, GR, IT, TR	AM	Stems, leaves
*Chaerophyllum bulbosum* L.	AM, BG, GR, IT, TR	AM	Stems, leaves, roots
		BG	Young shoots, corms, salad raw and stew
*Chaerophyllum macropodum* Boiss.	TR	TR	Eaten fresh
*Chaerophyllum macrospermum* (Spengel) Fisch. & C. A. Mey.	AM, TR	TR	Cooked as a stew or egg-vegetable dish, used in cheese production
*Conopodium majus* (Gouan) Loret	ES, IT, Sicily	ES	Tubers, raw as a snack (they taste like chestnut)
*Conopodium marianum* Lange	ES, MA	ES	Tubers, raw as a snack
*Conopodium pyrenaeum* (Loisel.) Miégev	ES, MA	ES	Tubers, raw as a snack
*Conopodium subcarneum* (Boiss. & Reut.) Boiss. & Reut.	ES	* ES	Tubers, raw as a snack
*Conopodium thalictrifolium* (Boiss.) Calest.	ES	* ES	Tubers, raw as a snack
*Coriandrum sativum* L.	native to JO, SY, naturalized to IT, MA, casual to ES, Sicily; introduced to AL, AM, BG, GR, Crete, CY, TR	AM	Stems, fruits
		TR	Cooked as a stew or rice-vegetable dish, pie is made from its seeds
*Crithmum maritimum* L.	BG, Crete, CY, ES, GR, IT, JO, MA, Sicily, SY, TR	IT	Young leaves, shoots boiled and dressed with vinegar (or lemon juice) and olive oil
		MA	Aerial parts
		* ES	Leaves, for seasoning olives; raw in salads; roots, raw as a snack
		CY	Young aerial parts, boiled, then pickled
		GR	Young aerial parts, boiled, then pickled
*Cuminum cyminum* L.	AM, ES, JO, casual to Sicily and MA	JO	Seed
*Daucus carota* L.	AL, AM, BG, Crete, CY, ES, GR, IT, JO, MA, Sicily, SY, TR	CY	Young aerial parts, boiled
		GR	Young aerial parts, boiled
		IT	Root, roasted, boiled, fried
		TR	Shoot and leaf meal, pancake, stew
		Crete	Young aerial parts, boiled in mixtures
*Daucus carota* L. subsp. *maximus* (Desf.) Ball	AL, BG, Crete, CY, ES, GR, IT, JO, MA, Sicily, SY, TR	JO	Fleshy roots, eaten raw
		* ES	Roots, as a snack, young leaves stewed
*Daucus carota* L. subsp*. carota*	AL, BG, Crete, CY, ES, GR, IT, MA, Sicily, TR	Sicily	Tender basal leaves and roots, raw in salads or stewed
*Deverra scoparia* Coss. & Durieu	MA	MA	Shoots, core of roots, aerial parts
*Deverra denudata* (Viv.) Pfisterer & Podlech	MA	MA	Shoots, core of roots
*Deverra tortuosa* (Desf.) DC.	JO	JO	Leaf and flower
*Diplotaenia turcica* Pimenov & Kljuykov (syn. ** *Diplotaenia cachrydifolia* sensu P. H. Davis, non Boiss)	TR	TR	Cooked as a stew or egg-vegetable dish; used in brine and cheese production
*Echinophora tenuifolia* L.	AM, BG, Crete, CY, GR, IT, Sicily, TR	TR	Shoot, flower, leaf soup, seasoning, drinking
*Elaeoselinum asclepium* (L.) Bertol.	AL, Crete, GR, ES, IT, MA, Sicily, TR	MA	Aerial parts
*Eryngium billardieri* F. Delaroche.	TR, AM, SY	TR	Eaten fresh
*Eryngium campestre* L.	AL, AM, BG, Crete, ES, GR, IT, MA, Sicily, SY, TR	AM	Leaves, roots
		Sicily	Sprouts, raw in salads
		TR	Shoot and leaf pie, stew
*Eryngium creticum* Lam.	AL, BG, Crete, CY, GR, JO, SY, TR, casual to IT	JO	Leaves and stems, eaten raw as salad with garlic and yogurt, or cooked as pastry
		SY	Young aerial part, salad, steamed
		Crete,	Young aerial part, salad
*Eryngium glomeratum* Lam.	Crete, GR, JO, SY, TR	JO	Young aerial part, salad
		Crete,	Young aerial part, salad
*Falcaria vulgaris* Bernh.	AL, AM, BG, ES, GR, IT, JO, SY TR,	TR	Aerial parts cooked as vegetable, leaves eaten in salads
		AM	Stems
*Ferula assa-foetida* L.	native to Libya	JO	Root
*Ferula communis* L.	AL, Crete, CY, ES, GR, IT, JO, MA, Sicily, SY, TR	IT	Inflorescences deep fried
		MA	Unopen inflorescence
		JO	Vegetables inflorescence, eaten cooked
*Ferula orientalis* L.	AM, BG, TR	TR	Cooked and consumed with yogurt; used in brine and cheese production
*Ferula rigidula* DC.	AM, TR	TR	Used in cheese production
*Ferulago angulata* (Schlecht.) Boiss. *subsp. angulata*	TR	TR	Used in cheese production
*Ferulago angulata* (Schlecht.) Boiss. subsp. *carduchorum* (Boiss. & Hausskn.) Chamberlain	TR	TR	Used in cheese production
*Ferulago setifolia* K. Koch	AM, TR	AM	Leaves, stems
*Ferulago stellata* Boiss.	TR	TR	Cooked as a stew or egg-vegetable dish, used in brine and cheese production
*Foeniculum vulgare* Mill.	AL, AM, BG, Crete, CY, ES, GR, IT, JO, MA, Sicily, SY TR	AM	Stems, fruits
		BG	Leaves, mericarps, spice
		CY	Leaves and tender stems, raw as a snack or in salads, or stewed and cooked, seasoning (especially snails)
		ES	Leaves and tender stems, raw as a snack or in salads, or stewed
		GR	Leaves and tender stems, raw as a snack or in salads, or stewed, fried, sauté
		MA	Tender stems, tender leaves, peeled roots, seeds
		TR	Aboveground meal, roasted, pilaf, as spice
		JO	“Seeds”, as herbal tea, ground “seeds” and added to bread loaves, dried “seeds” added to pickles, fruits foliage, dried foliage added to cake as a condiment, foliage, eaten raw as salad, or cooked as soup
		Sicily	Leaves and tender shoots, raw as a snack or in salads, or stewed, seeds as a condiment and for the herbal teas and digestive liquors preparation
		Crete	Leaves, fried in mixtures as a filling for pies or seasoning stewed potatoes and tomatoes
		SY	Aerial part, spice added to “zaatar” and soups, fried with eggs/meat/potato
*Foeniculum vulgare* subsp. *piperitum* (C. Presl.) Bég.		AL	Young aerial parts and fruits boiled, then on pasta or in mixed vegetables young leaves, shoots boiled and dressed with vinegar (or lemon juice) and olive oil
		IT	Young aerial parts and fruits boiled, then on pasta or in mixed vegetables young leaves, shoots boiled and dressed with vinegar (or lemon juice) and olive oil
*Grammosciadium platycarpum* Boiss. & Hausskn.	TR, AL	TR	Eaten fresh
*Heliosciadium nodiflorum* (L.) W.D.J. Koch (** syn. *Apium nodiflorum* (L.) Lag.)	AL, BG, Crete, CY, ES, GR, IT, JO, MA, Sicily, SY, TR	AL	Aerial parts raw in salads, seasoning soups
		IT	Aerial parts raw in salads, seasoning soups
		ES	Tender leaves and stems, raw in salads
		Sicily	Tender leaves and stems, raw in salads or stewed
		SY	Young aerial part, appetizer
		MA	Aerial parts
*Heracleum antasiaticum* Manden.	AM, TR	AM	Stems
*Heracleum persicum* Fisch.	TR	TR	Used in cheese production
*Heracleum sibiricum* L.	BG, IT, Sicily	BG	Leaves, shoots, salad raw, soup, spice
*Heracleum trachyloma* Fisch. & C.A. Mey.	TR, AM	TR	Leaves stuffed, stems as spice, eaten raw after bark is peeled
*Kundmannia sicula* (L.) DC.	Crete, ES, GR, IT, MA, Sicily,	Sicily	Basal leaves, stewed
*Levisticum officinale* W. D. J. Koch	native to JO; introduced to BG, ES; casual to AL. naturalized in IT	JO	Leaves
*Oenanthe pimpinelloides* L.	AL, BG, Crete, ES, GR, IT, JO, MA, Sicily, SY, TR	Crete	Young aerial parts, fried in mixtures in pies
*Orlaya daucoides* (L.) Greuter (syn. ** *Caucalis daucoides* L.)	AL, AM, BG, Crete, CY, ES, GR, IT, JO, MA, Sicily, SY, TR	AM	Stems
*Pastinaca sativa* L.	AL, AM, BG, ES, GR, IT, Sicily, TR	JO	Leaves, root
*Petroselinum crispum* (Mill.) Fuss	native to JO, MA casual to Sicily; doubtfully native to AL, introduced to AM, BG, GR, Crete, CY, TR; naturalized to ES	JO	Petiole, root, stem, leaves
*Pimpinella kotschyana* Boiss.	SY, TR,	TR	Used in rennet production
*Pimpinella saxifraga* L.	AL, AM, BG, GR, ES, IT, JO, SY, TR	AM	Seeds
		BG	Root, leaves, fruits, salad raw, spice
*Pimpinella cretica* Poir.	Crete, CY, GR, JO, SY, TR	Crete	Young aerial parts, in mixtures for pies
*Pimpinella peregrina* L.	AL, AM, BG, Crete, CY, GR, IT, JO, Sicily, SY, TR	Crete	Young aerial parts, in mixtures for pies
*Prangos ferulacea* (L.) Lindl.	AL, AM, BG, GR, IT, JO, Sicily, SY, TR,	AM	Leaves
*Pseudopimpinella anthriscoides* (Boiss.) F. Ghahrem. & al. (syn. ** *Pimpinella anthriscoides* Boiss.)	AM, SY, TR	TR	As spice, cooked as a stew or egg vegetable dish
*Ridolfia segetum* (Guss.) Moris	AL, Crete, CY, ES, GR, IT, JO, MA, Sicily, SY, TR	MA	Stems
*Scandix pecten-veneris* L.	AL, AM, BG, Crete, CY, ES, GR, IT, JO, MA, Sicily, SY, TR	* ES	Young basal leaves, stewed
		MA	Basal leaves
		GR	Young leaves, soups
		SY	Young aerial part, steamed for “sleeg” (rice and chicken folk dish)
		Crete	Young aerial parts, fried in mixtures, as a filling for pies
*Sium sisarum* L. *var. lancifolium* (M. Bieb.) Thell.	native AM, BG, GR, SY, TR, casual to ES, IT	TR	Used in cheese production
*Smyrnium olusatrum* L.	AL, Crete, CY, ES, GR, IT, JO, MA, Sicily, SY, TR	TR	Eaten fresh
		ES	Tender leaves and stems, raw in salads or stewed
		MA	Stems, young shoots
		GR	Young shoots, boiled, pastry
		IT	Young shoots, boiled, pastry
*Smyrnium perfoliatum* L.	AL, AM, BG, CY, Crete, ES, GR, JO, IT, MA, Sicily, TR	Sicily	Tender leaves and stems, pickled in vinegar
		BG	Root, leaves, spice, soup
*Smyrnium rotundifolium* Mill.	AL, BG, Crete, CY, GR, IT, Sicily, TR	Sicily	Tender leaves and stems, pickled in vinegar
*Thapsia villosa L.*	ES, MA	MA	Peeled roots
*Tordylium apulum* L.	AL, BG, CY, Crete, GR, ES, IT, JO, Sicily, SY, TR	AL	Aerial part, boiled
		CY	Young aerial parts, seasoning
		Crete	Young aerial parts, in mixtures for pies
		GR	Basal rosettes, boiled and pastry

## Data Availability

Not applicable.
